# Using the Project INTEGRATE Framework in Practice in Central Coast, Australia

**DOI:** 10.5334/ijic.4624

**Published:** 2019-06-21

**Authors:** Donna M.Y. Read, Hazel Dalton, Angela Booth, Nick Goodwin, Anne Hendry, David Perkins

**Affiliations:** 1 Centre for Rural and Remote Mental Health, University of Newcastle, NSW, AU; 2 International Foundation for Integrated Care, Wolfson College, Oxford, UK; 3 International Centre for Integrated Care, University of the West of Scotland, UK

**Keywords:** integrated care, mixed methods, Project INTEGRATE, formative evaluation, Australia

## Abstract

**Introduction:**

Integrated care implies sustained change in complex systems and progress is not always linear or easy to assess. The Central Coast integrated Care Program (CCICP) was planned as a ten-year place-based system change. This paper reports the first formative evaluation to provide a detailed description of the implementation of the CCICP, after two years of activity, and the current progress towards integrated care.

**Theory and Methods:**

Progress towards integrated care achieved by the CCICP was evaluated using the Project INTEGRATE Framework data in a mixed methods approach included semi-structured interviews (n = 23) and Project INTEGRATE Framework based surveys (n = 27). All data collected involved key stakeholders, with close involvement in the program, self-reporting.

**Results:**

Progress has been mixed. Gains had most clearly been made in the areas of clinical and professional integration; specifically, relationship building and improved collaboration and cooperation between service providers. The areas of systemic and functional integration were least improved with funding uncertainty being an ongoing significant problem. The evaluation also showed that the Project INTEGRATE framework provided a consistent language for CCICP partners and for evaluators and consistent indicators of progress. The framework also helped to identify key facilitators and barriers.

**Discussion and Conclusion:**

The findings highlight the willingness and commitment of key staff but also the importance of leadership, good communication, relationship building, and cultural transformation.

## Introduction

Integrated care has become a popular strategy for improving health system performance [1]. Integrated care has come to mean care with a person-centred focus and a population health approach [2]. This implies service changes to ensure care is co-ordinated around the needs of the people involved (patient, carer, family member or population) to overcome fragmentation of service delivery and improve quality and efficiency [3]. Person-centred care means that health and social needs, and individual preferences and values are addressed [4], and anticipates consumers and carers will be actively involved in care decisions and planning [5]. Hence, integrated care aims to improve access, quality and continuity of treatment, reduce fragmentation of services and improve health outcomes (including consumer perceptions of outcomes) through service innovation [3,4].

Wherever the impetus for integrated care begins, it is widely recognised that health and wellbeing promotion, taking a population health and person-centred approach, needs to involve cross-sector collaboration. Health is heavily affected by social determinants and disadvantage results in poor health outcomes and health inequities [6,7,8]. Social determinants of health include, but are not limited to: the environments in which people live (e.g. affordable housing and safe neighbourhoods), their access to education and fair working conditions, and access to healthcare and social services throughout the life course [9].

Working against the implementation of integrated care initiatives is the distributed accountability for services across government departments and the fragmentation of services within healthcare systems across the developed world [10]. It is notable in particularly successful integrated care programs in Scotland and Canada that provision of health and social services are jointly administered [11,12].

In Australia, as in many countries, services with a major role to play in determining health outcomes (healthcare, social care and education) are administered by different government departments with disparate funding arrangements, goals and responsibilities. Moreover traditionally, there has been little interdepartmental collaboration. Furthermore, the healthcare context is particularly complicated. Services are delivered by various public and private providers which have differing goals and responsibilities. Funding may be by federal, state government or private entities. The Federal government oversees primary care services through direct payments to General Practitioners (GPs) and allied health professionals and support of locally planned and commissioned primary care initiatives via Primary Health Networks (PHNs). The state government funds state-run health services to deliver secondary and tertiary care in the community and hospital settings. Complexity and overlap result in services funded by one source having implications for services funded by another without common accountability to foster improvements in collaboration and efficiency. For example, downstream efficiencies incurred in state funded hospitals require upstream interventions by GPs and other health service providers in the community, many of whom are not accountable to state health authorities.

Moreover, fee-for-service funding arrangements which reward the quantity of services rather than the quality have generally been employed. As such these funding arrangements tend to overlook the population health needs and discourages collaboration to address service user needs and outcomes. Therefore, these funding arrangements may result in unintended penalties for good practice [13].

On the other hand, the public provision of universal healthcare under Medicare in Australia has achieved many care advances and an excellent public health service, via its public/private system [14]. Australian advances in care, population health and other areas of healthcare, over the last quarter-century have been noted in international comparisons [15].

Nevertheless, the effectiveness of healthcare in Australia is threatened by the lack of integration. Specifically these threats include: rising costs; problems with the structure and integration of the healthcare system; changing demographics and an ageing population; problems of equity; risks to the health of minorities and other groups; and issues with the efficiency and sustainability of the overall health system and its workforce [14]. Schneider et al. find that Australia performs badly in regards to equity of service provision compared to other high-income countries [15]. These problems need to be addressed if the public provision of healthcare is to be maintained at a high, sustainable and equitable standard. The equitable delivery of health services being an explicit aim of the Australian Government [16]. Similar issues also arise in the private provision of healthcare in Australia. Analogous problems have been noted in other developed healthcare systems – particularly in the United Kingdom and United States [15].

In order to advance sustainable and equitable health in New South Wales (NSW) a whole of government approach (i.e. government departments working together to achieve shared goals) to integrated care was proposed [17]. In line with this plan, NSW Health (i.e. the state government department) committed $180 million over 6 years (2014 to 2019) to an integrated care strategy, a key part of which were three demonstrator sites: Western NSW, Western Sydney and the Central Coast LHDs [18].

The demonstrator LHDs were expected to develop and scale up successful approaches to integrated care through the coordination of services appropriate to local needs. LHDs were expected to work in partnership with PHNs and other health agencies in the primary care, not-for-profit and private sectors and share the learnings so that successful approaches could be introduced throughout NSW [18].

In this paper, we present findings from a formative evaluation undertaken during 2017 of the Central Coast Integrated Care Program (CCICP), the name adopted by Central Coast Local Health District (CCLHD) for their work as a demonstrator site. The CCICP was undertaken in partnership with the Hunter New England and Central Coast Primary Health Network (HNECCPHN), Family and Community Services (FACS) NSW, NSW Ambulance and the NSW Department of Education [19]. The Central Coast of NSW lies just north of Sydney and covers 1681 km2 [20]. The CCLHD serves approximately 340,000 people [21]. The CCICP approach sought to provide integrated health and social care underpinned by the concepts of inter-organisational partnership, risk stratification and commissioning. The identified key health needs were the increasing emergency department attendances and hospital admissions particularly by the relatively high numbers of people with chronic and complex conditions, older people and vulnerable children and young people [22]. This paper describes the progress made by the CCICP toward integration using the Project INTEGRATE Framework. For a detailed description of the CCICP see Dalton et al. [22]. This is the first time that the Project INTEGRATE Framework has been used in a formative evaluation and applied in the Australian context. Therefore this paper contributes to knowledge through the testing of a novel approach to evaluating integrated care progress.

## Theory and methods

According to Stetler et al. (2006) a formative evaluation is: “a rigorous assessment process designed to identify potential and actual influences on the progress and effectiveness of implementation efforts” [23 p. S1]. Thus, the overall aim of the evaluation was to provide a detailed description of the implementation of the CCICP, and its effect on progress towards integrated care, in the context of Central Coast strategies and priorities [24].

A co-design approach to the evaluation was taken. Members of the research team facilitated two initial workshops with the CCICP key partners in February 2017. These workshops assisted the research team to understand the CCICP and the significant events and activities that affected its progress and trajectory. In these workshops, the availability of data was explored and a pragmatic and informative methodology agreed upon. It was decided that the Project INTEGRATE Framework should be employed to enable comparison to international benchmarks which facilitated identification of strengths, weaknesses and gaps. Here we focus on the findings specifically pertaining to the use of the framework, addressing the research question: What progress has been made by the CCICP towards integration of care?

### Project Integrate

**Figure 1:**
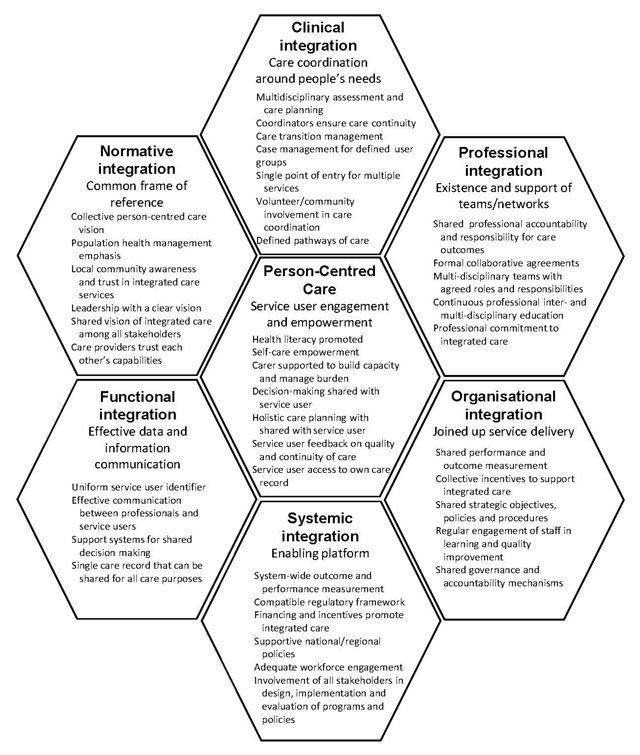
Project INTEGRATE Key Dimensions and sub-elements of Integrated Care [5].

The development of the Project INTEGRATE Framework began with a comprehensive, literature review which focused on conceptual frameworks or relevant aspects explaining integrated care, which identified 18 frameworks, which were subsequently analysed and a new framework distilled. Then the framework was validated for contextual independence, that is, it would apply in differing health system environments and across target populations or groups [25]. The seven dimensions are consistent with the with Valentijn et al.’s conceptual model now known as the Rainbow Model of Integrated Care [26]. The framework elucidates best practice by identifying the sub-elements found to be required for successful implementation through evidence obtained from a four-year study of integrated care in Europe [5,25,27]. The dimensions and sub-elements (See Figure 1) are considered appropriate for successful integrated care implementation across countries and population groups regardless of the health condition or care group issue. Hence, the framework enables reflection on the design and implementation of integrated care programs and comparison to other international initiatives [5].

### Data collection and analysis

A mixed methods approach was chosen with three methods of data collection: review of 25 project documents related to the activities of the CCICP from 2014 to 2017; an online survey of a broad group of key stakeholders; and in-depth semi-structured interviews with a subset of these stakeholders. The CCICP key partners identified the key stakeholders to be invited (both survey and interview samples). The criteria they applied for sample inclusion were representation across the breadth of the program (CCICP overall, individual streams, key projects identified for deeper review), representation across the depth of the program (from operational on the ground workforce through to leadership and executive sponsoring roles), all had close involvement in, and knowledge of, the program. In addition, a third workshop with the CCICP key partners in August 2017 conducted situational analysis of CCICP progress, examined interim survey results reviewed and reflected upon evaluation outcomes to date. The progress towards integrated care was primarily assessed through survey and interview data. The workshops and document review had most relevance to the implementation description.

The survey was developed for this evaluation, with questions structured around the Project INTEGRATE Framework. Participants were asked whether each sub-element was being implemented in the CCICP (using a 5-point Likert scale: strongly agree, agree, neither agree or disagree, disagree, strongly disagree). For example in relation to person-centred care: ‘Service users and care professionals work together to obtain and understand basic health information to make appropriate health decisions.’ Hence, there were eight questions related to patient-centred care, eight related to clinical integration, six related to professional integration, five related to organisational integration, seven related to systemic integration, and five to functional integration (39 questions in total). Participants were also invited to provide additional commentary in open-text boxes. Since normative integration was not included in the original description of the framework [25], this dimension was not included in the survey. No demographic data or differentiating data was collected in order to protect anonymity within the small sample, and because the survey targeted only those with close involvement in the program, and there was no intention to analyse by sub-set.

The survey was administered online (using the Research Electronic Data Capture (REDCap) platform (www.project.redcap.org)) between August and September 2017. A total of 60 stakeholders (representatives from CCLHD, HNECCPHN, government agencies, non-government agencies, consumer and carer advocacy groups and GPs) were invited to participate and 27 surveys were completed (45% response rate). The majority of participants (67%) were from the CCLHD with fairly even representation from other invited organisations.

In-depth, semi-structured interviews were conducted either face-to-face or by telephone. The duration of the interviews ranged from 30 to 90 minutes; each was audio-recorded and transcribed verbatim. Interview questions were developed to gain an in-depth understanding of the participants’ experiences and their perspectives on the context, activities and progress of the CCICP. Questions asked about the participant’s perspective of: their role and how the program has affected the way they work, the meaning of integrated care, the program’s objectives, how the program was being implemented (including facilitators and barriers to implementation), the success of the program and its benefits, and the future of the program. A total of 51 stakeholders were invited and 23 participated in the interviews conducted in September and October 2017.

Simple descriptive statistics were used to analyse the quantitative survey data. Hence percentage of informants agreeing, disagreeing or with a non-committal response was calculated.

Qualitative data from the interviews and survey was first coded to the relevant Project INTEGRATE Framework dimension. Open coding then identified the themes within each dimension. This coding approach allows connate data to be gathered for interpretation [28,29]. NVivo software (https://www.qsrinternational.com/nvivo/home) was used to aid management and coding of data. The data was analysed by an experienced qualitative researcher whose PhD was entirely qualitative. Due to the need to meet deadlines set by commissioners of the work there was insufficient time for more than one researcher to code the data. However, the findings were sense checked with stakeholders.

Participation in surveys and interviews was voluntary. Where quotes are used in this paper, no identifying information is given to protect participants’ identity. Ethics approval for the evaluation was granted by the Hunter New England Human Research Ethics Committee (approval 17/06/21/5.01) and site-specific approval obtained. This ethics approval was recognised by the University if Newcastle (ref no. H-2017-0218).

## Findings

### The Central Coast Integrated Care Program

**Figure 2:**
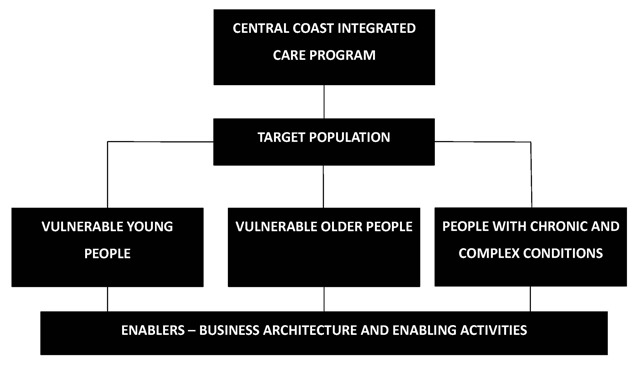
Central Coast Integrated Care Program Overview.

The original 10-year implementation plan for the CCICP was structured around three population streams with ‘enabler activities’ (see Table 1) supporting these streams (see Figure 2). A governance structure was established to guide and oversee the project with monthly governance meetings. Membership included Chief Executives of both the Central Coast LHD and the HNECC PHN, the district Director of FACS, several senior staff related to the CCICP and a GP representative. Regular governance reports were tabled at these meetings to track CCICP milestones and overall program progress. Sub-projects were subject to their own tracking reports and the CCICP also reported on measures mandated by NSW Health.

**Table 1:** Enabler activities supporting the population streams.

Enabler	Description

Population health approach	Vulnerable groups were identified by need, disadvantage, and likelihood to be high users of health services in the future. A risk stratification model, informed by a detailed diagnostic assessment, was carried out to identify the three target populations.
Outcomes-based commissioning	Outcomes-based commissioning places the emphasis on the achieving the desired outcomes for the service user (rather than the more usual measure of performance by activity). The CCICP tested outcomes-based commissioning in the context of NGO-provided care coordinators for a Central Coast sub-population of vulnerable older people (North Wyong region).
Co-design	Co-design, that is the involvement of stakeholders in service design, was used to varying degrees. For example, substantial consultation and workshops were undertaken for both care coordination and shared care planning in the vulnerable older people stream but less so in the chronic and complex stream. In the vulnerable youth and children stream, there was much engagement and dialogue with stakeholders and to a lesser extent with the young people involved.
Information sharing tools	A key enabler of integrating care is the ability to share information safely and securely amongst care professionals. A number of projects to improve information sharing, identifying, selecting and enabling a shared care planning system were undertaken. Several options were considered but an IT platform that could deliver all of these needs has not yet been identified. Shared care planning work was deferred in order to prioritise work on other objectives.
Multiagency Accelerated Implementation Methodology (AIM)	A lack of workforce change management skills, lack of a common language across partner organisations and professionals, and resistance to change were identified as key barriers to successful program implementation. An evidence review identified that joint training in the use of a consistent framework and change management approach would support effective interagency work and therefore Aim was trialled. AIM is an internationally recognised change management methodology supported by the Agency for Clinical Innovation (ACI) and the Health Education and Training Institute (HETI) for NSW Health staff to practically assist with project implementation. In order to build capacity to deliver collaborative change 97 staff trained in 2016. Importantly, the two-day training sessions were delivered purposefully as cross-sectoral training to groups containing a mix of LHD and partner agency staff, including HNECC PHN, FACS, DEC, NSW Ambulance and the Family Referral Service (the Benevolent Society). Feedback from the training was overwhelmingly positive and further training was delivered in 2017 and planned for 2018.
International evidence and experts	Evidence and international experts in integrated care to inform planning, implementation and review of progress have been drawn upon since inception.

The three population streams were vulnerable youth and children, vulnerable older people and people with chronic and complex conditions. The vulnerable youth and children stream focused on working with key partner agencies (HNECCPHN, NSW Department of Education, and FACS) through schools. This included three important initiatives: Family Referral Service in Schools (an early intervention program working with children and their families to address health and social vulnerabilities by connecting with services and supporting children to engage in learning); Central Coast Multi-Agency Response Centre (CCMARC) (Centre in which FACS, the LHD and other organisations are collocated to facilitate information sharing to enable early intervention responses for children at significant risk of harm); and Out Of Home Care Health Access (an integrated, multi-agency response to improve service delivery around health needs for children and youth in out of home care). The vulnerable older people stream focused on a co-designed, outcomes-based commissioning project. The chronic and complex conditions stream built on, aligned with and consolidated a number of existing projects that were amenable to inclusion in the CCICP. Notably these included, the Central Coast Alternative Pathways Initiative (CCAPI), a new care model allowing ambulance drivers responding to emergency calls to refer low acuity cases to usual care providers or available general practices, rather than conveying to emergency departments as the default option. In addition, the Chronic Disease Management Program was redesigned to refocus the model of care in general practice, known as the Woy Woy Integrated Care Pilot (WWICP).

It should be noted that the context in which CCICP was implemented changed over the period of planning and implementation and these events affected the trajectory and tempo of implementation. First, the appointment of the full CCICP team was not completed until April 2015 which could therefore be considered as the true start date. Secondly, the health sector underwent major restructuring. Most notably in July 2015 an original CCICP partner, the Central Coast Medicare Local, was replaced by the HNECCPHN. This replacement resulted in a renegotiation and a change in focus; broadening scope from primary care and general practice support to a commissioning function and work in low and moderate intensity mental health services and suicide prevention. There were also staffing changes and loss of key contacts and historical knowledge in FACS due to a restructure in September 2016. This slowed the momentum of the work with this partner. Further, the CCLHD leadership had also undergone changes at the CEO level in August 2016 and the CCICP leader changing in February 2017. Leadership changes also occurred at the State level. February 2017 saw the retirement of the Health Minister Jillian Skinner who was replaced by Minister Hazzard. There have also been uncertainties, particularly in the lead up to the evaluation, around the ongoing funding of the CCICP.

### Perceptions of progress towards integrated care

Most strikingly the survey results (see Table 2) show that there was a great deal of uncertainty about progress towards integrated care. More participants responded that they neither agreed nor disagreed than agreed with achievement in several sub-elements representing all dimensions of integration (person-centred care (2 sub-elements), clinical integration (3 sub-elements), professional integration (1 sub-element), organisational integration (1 sub-element), systemic integration (3 sub-elements) and functional integration (1 sub-element). Some participants identified small improvements, although others perceived that little in practice had changed. The general finding was that more needed to be done.

**Table 2:** Survey results summary.

Dimension	Responses agreeing with statement of best practice	Strongly agreed/agreed	Neither agreed or disagreed	Disagreed/strongly disagreed	Summary analysis of free text

Patient-centred care	CCICP contributed to improved achievement in patient-centred care	82%	19%		Uncertainty about achievements Limited improvements Policy intent not always translated into practice Much more needs to be done (n = 13)
	Health literacy promotion	70%	26%	4%	
	Decision making shared with service user	59%	33%	7%	
	Self-care empowerment	52%	44%	4%	
	Carer support	44%	56%		
	Service user feedback on service	44%	37%	19%	
	Holistic care planning shared with service user	41%	48%	11%	
	Access to own care record	41%	37%	22%	
Clinical integration	CCICP contributed to improved achievement in clinical integration	82%	7%	11%	CCICP has had positive impact in places Lack of perceived change Limited improvements Much more needs to be done (n = 14)
	Multidisciplinary assessment and care planning	78%	7%	15%	
	Coordinators ensure care continuity	59%	37%	4%	
	Case management of defined user groups	59%	37%	4%	
	Single entry point for multiple services	59%	15%	26%	
	Care transition management	37%	52%	11%	
	Defined pathways of care	30%	59%	11%	
	Volunteers/community actively involved in coordinating care	15%	67%	19%	
Professional integration	CCICP contributed to improved achievement in professional integration	74%	11%	15%	Primary health care often omitted from teams Little evidence of multi-professional education Ideological rather than actual commitment Lack of perceived change Much more needs to be done (n = 13)
	Shared professional responsibility and accountability	70%	26%	4%	
	Multi-disciplinary teams with agreed roles and responsibilities	67%	26%	7%	
	Professional commitment to integrated care	63%	11%	15%	
	Formal agreements support professional collaboration	56%	41%	4%	
	Continuous multi- and inter-professional education	37%	56%	7%	
Organisational integration	CCICP contributed to improved achievement in organisational integration	74%	15%	11%	Uncertainty about achievements Limited improvements Variable across projects Lack of perceived change Much more needs to be done (n = 12)
	Shared performance and outcome measurement	56%	33%	11%	
	Collective incentives	56%	37%	7%	
	Shared strategic objectives, policies and procedures	52%	33%	15%	
	Shared governance and accountability mechanisms	41%	56%	4%	
Systemic integration	CCICP contributed to improved achievement in systemic integration	67%	26%	7%	Lack of perceived change Uncertainty about achievements Gap in workforce numbers, skills and competencies Lack of authorization to express ideas Lack of perceived change State and national policy constraints Much more needs to be done (n = 11)
	Involvement of all stakeholders in design, implementation and evaluation of programs and policies	44%	26%	30%	
	Supportive national/regional policies	37%	37%	26%	
	Compatible regulatory framework	33%	44%	22%	
	Financing and incentives promote integrated care	33%	48%	19%	
	System-wide outcome and performance measurement	30%	52%	19%	
	Investment in an adequate workforce	19%	33%	48%	
Functional integration	CCICP contributed to improved achievement in functional integration	44%	44%	11%	Lack of perceived change Uncertainty about achievements Poor communication Lack of electronic systems for sharing data, sharing care planning and making referrals to all partners Much more needs to be done (n = 13)
	Uniform service user identifier	37%	26%	37%	
	Support systems for shared decision making	26%	41%	33%	
	Effective communication between professionals and service users	19%	52%	30%	
	Single care record that can be shared for all care purposes	15%	33%	52%	

There was strongest agreement that gains were being made in the sub-elements of professional integration. However, most participants were uncertain that continuous multi and inter-professional education was being supported. The only sub-element, apart from professional integration, for which there was more than two-thirds agreement was health literacy (professionals assisting service users to obtain and understand health information to aid their decision making – person-centred care). Furthermore, there was widespread of agreement that the CCICP had contributed to progress in patient-centred care and clinical, professional, organisational and systemic integration. The advancement of functional integration was the exception where less than half of participants agreed the CCICP had contributed.

The only sub-elements where more participants disagreed they were being achieved than agreed were Investment in an adequate workforce (systemic integration) and shared care records (a single health record to allow data sharing for multiple purposes – functional integration).

The interview narratives generally support the survey findings concerning a lack of clarity about what was being achieved, what was being implemented and that improvements were limited. Participants provided some examples of achievements but also indicated there was much more work to be done. For example, it was clear that service providers were working together in all three streams reflecting aspects of clinical, professional and organisational integration. Extremely apparent in nearly all interviews was the participants’ commitment to integrated care partnerships. They expressed not only their own commitment but that of their co-workers. One participant said:

“I want to see the organisation go in an integrated care direction. I am hugely committed to that. I think that is the way to go. I think that’s where we’re going to get our best benefit for our community.”

However, shared-care planning appeared to be more about clinical integration than involving the service user in their care. One participant from the youth and children stream said:

“I think there’s still a level of bravery that our service providers need to take around really sharing the care, because they’re sharing the care with each other a little bit better at times, when it’s pointy end but they’re not sharing the care I don’t think enough with our parents and our students.”

Gains in systemic and functional integration were less evident. Importantly, however, interview participants involved in the youth and children stream referred to the recognition of key privacy legislation (16A of the Children and Young Persons’ Care and Protection Act 1998, 2009 amendment) that had made sharing of data possible.

Organisational, systemic and functional integration gaps were particularly evident in the interview narratives. While overall narratives indicated that service providers were often working under agreed protocols or guidelines, the systems needed to enable clinical and professional integration were generally referred to as being developed. For example, a participant described barriers that still needed to be overcome for a central intake system to aid referral pathways:

“It’s in progress, and I think there’s cultural issues, there’s logistical space issues, being able to house everybody you need for intake. There’s cultural issues in terms of people accepting that’s what it is. …we’ve traditionally done our referrals to each individual service and they’ve done by the same person who provides the service… I think we’ve gone to try to do centralised intake three times in my time with chronic disease programs. And we get to a certain point that we never quite get there. So we’re further along this time around under the integrated care, but it’s still not quite there.”

Furthermore, while there was an overall Memorandum of Understanding, some participants indicated that there was uncertainty about what the organisations involved had agreed to in practice. In relation to CC MARC (see Table 1) a participant said:

“…our leaders agreed to it but they didn’t agree to what they’d agreed on and no one was there to witness the agreement. It was verbal. It wasn’t concrete. There wasn’t clear governance around it. It was a troublesome way to do business. We kind of had to make it work and we found ways to try to join, but we also learnt a lot about how hard it is when you force people into a room. You do a project but they’re not sure what problem they’re trying to solve.”

Looking to the future, numerous participants reflected on future plans to extend the partnerships into a more formal alliance which would improve organisational integration, with the initial focus on the work between the health district and the primary health network. One participant acknowledged the evolution of the partnered working:

“…we had some strong relationships, we had some MOUs. We’re now moving perhaps to a more sophisticated and mature relationship where we actually have this alliance where we’re – you know, not only doing our business, but we’re making sure that your business is going okay, and if it’s not, how do we support you to achieve your objectives.”

Communication was highlighted as an area that needed improvement. The incompatibility of IT systems was raised as a major issue hampering the progress towards integrated care. Despite some attempts at this problem, the issue had not been overcome. However more broadly, communication was seen as something that had not been done well without a communication plan (steps were in place at the time of interview to address this):

“So just down to communication, another big thing. We didn’t do communication well …we didn’t have a clear communication plan to begin with.”

Moreover, participants suggested different workplace cultures fostered a lack of common understanding despite AIM training (see Table 1). It should be noted that AIM training gave participants a common language for change management, crucial for implementation of the CCICP. The deliberate act of training different service partners together fostered their relationships and common understanding of change management, however this remains distinct from a common language for integrated care. At the third workshop, core CCICP participants reflected positively on the use of the framework, which challenged the group to reflect on progress and inform future plans. The framework was perceived to give a structured common language for integrated care and aided in the identification of strengths, weaknesses and gaps.

More broadly, how the health system and other government ministries operated and were funded was generally seen as being contrary to integrated care. Participants discussed short-term funding of services, short-term staff contracts, fragmented commissioning of services and funding arrangements that discouraged cooperative working arrangements.

The only data of normative integration came from interviews which provided evidence for some progress towards developing a collective vision of person-centred, population health based approach. Nevertheless, while most participants said integrated care meant person-centred care this did not necessarily translate to service users being included in their care as discussed above.

There were mixed reports on the ability of leadership to maintain the collective vision. The vision and commitment of project leaders and team members and key organisation leaders was often noted. The support given to the people they were working with was seen as key to progress:

“I guess, that commitment at a really senior level. So having the CEO and the DD [District Director] and, you know, all of those kind of people commit to a bit of a vision at the start and then freeing up or facilitating their resources to be able and participate. That real high-level commitment type stuff and then prioritising it through line agencies certainly helped.”

Conversely, participants reported aspects of leadership that had been missing over the course of the project. As discussed above there were failures to clarify working arrangements. Furthermore, senior management of the key organisations, including the NSW Ministry of Health, changed. It was considered by four participants that the strong leadership that they had appreciated was lost. The new organisation managers and the Ministry were considered to be showing less vision and commitment and to be more risk adverse:

“So I think we have a manager. We don’t necessarily have a leader at this point in time… I think lack of leadership, strong leadership upwards – it’s made it difficult, and associated with quite a constrained view of what integrated care is.”

Those in current leadership positions were understood to be less willing and able to negotiate a frontier pushing agenda. These reflections were gathered in a period of funding uncertainty. On the other hand, it was also noted that the new leadership had not been in place long enough to rebuild relationships.

It was apparent in interviews that there had been a failure to create a common vision, readiness and commitment. Resistance among some service providers to the changes needed was noted. A participant in the vulnerable older people stream said:

“…everybody is so guarded too. We’re all guarded. Well, that’s my space. You’re saying my work is no good, so I think they had to combat that, which would have been really hard.”

## Discussion

The evaluation of the CCICP using the Project INTEGRATE framework presents an overall picture of limited gains. These gains have mostly been in the areas of person-centred care, clinical integration, professional and organisational care. Gains have most clearly been made in the area of partnering and building of relationships between organisations that have led to improved collaboration and cooperation between service providers. Moreover, stakeholders demonstrated considerable commitment to the integrated care goal. As the formative evaluation was carried out at two years into a 10-year strategy achievements being limited should not be surprising. Rather they indicate that progress has been encouraging.

Nevertheless, the evaluation also identifies areas where the CCICP should pay attention. Least appeared to be being achieved in the areas of systemic and functional integration. The most notable gaps identified are in the areas of routinely involving service users in their care plans, ensuring the buy-in of service provider staff and a common system for sharing of service user information electronically. Furthermore, funding uncertainty was also identified as a significant problem area with the continued practice of short-term, fragmented commissioning of services.

The struggle apparent in the CCICP story has been similarly noted in accounts of European initiatives [10]. In Europe it was noted that a stable policy was a requirement for sustainability of initiatives [10]. The CCICP has had to contend with great shifts in policy and organisational structure context. Moreover, the areas where the CCICP are struggling the most are not simple to overcome. Reorienting service provision so that service users are central rather than service providers is likely to require systemic changes [1]. Berwick [30] argues that in the USA vested interests, that view healthcare as a money making venture act to resist change. This is likely to apply to the Australian context as well since in common with USA, Australia has a mix of private and public health funders. Moreover, a shared funding model has been identified as a key measure for facilitating integrated care [10,31]. This measure is less likely to gain support in the CCICP context; where the financial bottom-line is the major consideration. Nonetheless, future planning for the CCICP pertained to a formalised alliance, which may edge closer to a shared funding model.

Although the CCICP made good advances to achieve the normative changes needed at the beginning, some of the impetus appears to have been lost. This hiatus in progress appears to have been associated with changes in leadership within the project, the organisations involved and the NSW Ministry of Health. The changes in leadership (including at the CCICP, CCLHD, and state health minister level) were perceived to have resulted in a narrowing of the vision for the CCICP and hence the ability to take “big innovative steps” considered necessary [1]. This was coupled with a window of funding instability for the CCICP. This evaluation supports Amelung et al.’s perception that leadership capable of fostering an innovative environment and a shared vision between the disparate players involved in delivering integrated care is of vital importance [32]. Strong leadership is needed to bring about changes in culture (“the way we do things around here” [33, p.237]) from a number of professions and organisations. Professional and organisational cultures tend to be entrenched and not easily changed [34]. The commitment shown by participants is likely to be a vital part of bringing about the cultural changes needed to ensure the buy-in of all staff. Leadership can occur at any level of the hierarchy [32]. Furthermore, time is essential for the building of trust and relationships necessary for service providers to work together [10] and for adjustments to a new way of doing things to become normalised [32]. This evaluation comes only after two years of implementation.

A key and related finding is the gap in communication with participants. Communication is an area often given insufficient attention in integrated care initiatives and is vital for ensuring the development of relationships and a shared vision [35]. While the Project INTEGRATE framework appeared to provide concepts around which service providers could build a common understanding of integrated care it cannot be assumed that this will lead to improvements in communication with service users. Indeed in this evaluation, communication with service users was identified as an area in particular need of improvement, both in relation to their own care and in regard to service development. Ultimately, integrated care is about delivering the outcomes and services that the service user wants or needs [1]. Furthermore, successful models demonstrate the importance of delivering person-centred care and involving service users in the development of services [e.g. 12,36,37].

A last reflection on the use of the Project INTEGRATE framework and the co-design approach taken with this formative evaluation is that the workshops (initial co-design, situational analysis of CCICP progress using the Project INTEGRATE survey results, and the final presentation of the formative evaluation) conducted with the key stakeholders were perceived by them as opportunities to reflect on progress and enable strategic decision making at those times, thus value and progress was made prior to the finalisation of the formative evaluation.

## Limitations

The main limitation of the evaluation is that it relies on the perspectives of a small number of stakeholders who had a great deal of involvement in the CCICP. Given the participants involved, and that data collection relied on self-reporting, the presentation of a favourable picture is perhaps unsurprising. Nevertheless, despite this potential for bias, participants readily described areas where problems were being faced. A further evaluation more objectively measuring impacts would help to confirm the findings here. It would also be desirable to include the perspective of service users in any future evaluation.

In addition, the appropriateness of the Project INTEGRATE framework has not been tested in the Australian context previously. Nevertheless, the Project INTEGRATE framework provided consistent indicators of progress and a consistent language for the discussion of the evaluation with CCICP service providers. The framework also helped to identify key facilitators and barriers. While the study confirms the usefulness of this tool for exploring integrated care progress we did encounter some challenges using the framework. Discussion of these challenges are beyond the scope of this paper.

## Conclusion

The evaluation demonstrates that the CCICP has achieved some early gains towards integrated care. It is apparent that because of the willingness of those involved, collaborative approaches to service provision have been achieved despite lack of strong systemic and functional supports. The findings also reflect the difficulties of trying to orchestrate the complex changes needed for implementation of integrated care in a shifting context beyond the control of the organisations involved. The inclusion of more voices, including service users, would be considered in future evaluations.

Furthermore, the importance of leadership, good communication, relationship building and cultural transformation has been highlighted. Looking forward, to maintain the momentum of what has already achieved it will be vital to ensure leadership able to: communicate a shared vision, build and promote relationships and engage the workforce in the task, and thereby build an appropriate cultural environment. The lack of buy-in by some staff suggests that there is a need for greater attention to what the staff need in order for them to deliver the services required. Furthermore, research that investigates how implementing an integrated care program affects staff is warranted.

Nevertheless, a further lesson learned is that while waiting for organisational and systemic supports, advancements towards integrated care can be made largely through the efforts and goodwill of frontline staff. However, success in integrated care is unlikely to be sustained without those functional, organisational and systemic supports. We would argue those higher order changes are also important for normative change which promotes a virtuous cycle of commitment to integrated care.
